# Dosimetric robustness of urethra sparing in ultrahypofractionated prostate SBRT using catheter-based motion tracking

**DOI:** 10.1007/s00066-026-02547-8

**Published:** 2026-05-22

**Authors:** J. Berchtold, M. Schichl, N. Annon-Eberharter, I. M. Messner, C. Gaisberger, F. Röder, F. Wolf

**Affiliations:** 1 Dpt. of Radiation Oncology, Paracelsus Medical University of Salzburg, Müllner Hauptstraße 48, 5020 Salzburg, Austria; 2 RadART - Institute for Research and Development on Advanced Radiation Technologies, Paracelsus Medical University, Müllner Hauptstraße 48, 5020 Salzburg, Austria

**Keywords:** UHFX, Intrafraction IGRT, Motion management, Radiotherapy, RayPilot Hypocath

## Abstract

**Purpose:**

In ultrahypofractionated prostate stereotactic body radiation therapy (SBRT), genitourinary toxicity is the primary concern. Urethra sparing is a strategy to mitigate potential negative effects of higher single doses to the urethra and bladder neck. We report on the dosimetric robustness of urethra sparing using a catheter-based real-time monitoring device to track both interfractional variability and intrafractional motion.

**Methods:**

In 12 patients undergoing prostate ultrahypofractionated radiotherapy (UHFX), intrafractional prostate motion was monitored using a HypoCath catheter (Micropos Medical™, Gothenburg, Sweden), enabling urethra contouring on the planning CT and cone-beam (CB)CT images used for image-guided radiation therapy (IGRT). Dose coverage of both the urethra and the prostate clinical target volume (CTV) was calculated and compared between the planning CT and matched CBCTs. In addition, prostate motion was simulated and the cumulative dose coverage of prostate and urethra was calculated as a function of time.

**Results:**

The deviation between the planned and delivered urethral dose strongly correlated with elapsed time. Variations in the urethral curvature between planning CT and CBCT had only a minimal effect on the dose distribution. The combined effects of deviations in the urethral shape and prostate movement resulted in a mean dose deviation of 76 cGy for the urethral D1 of all fractions and patients. The patient who experienced the greatest prostate motion showed an increase in urethral D1 of 1.94 Gy (+5.2%).

**Conclusion:**

Urethra sparing is feasible and only marginally impaired by variations in urethral shape but is sensitive to longer treatment durations due to intrafractional movement. Direct visualization of the urethra is not essential for IGRT, but intrafractional motion control is recommended.

## Introduction

Ultrahypofractionated radiotherapy (UHFX) has become a treatment alternative for localized prostate cancer, offering improved convenience and potential radiobiological advantages.

The rationale for sparing the urethra and bladder neck arises from the observation that genitourinary (GU) toxicity appears to be increased and may be dose limiting in UHFX of the prostate. Recent large, randomized trials comparing UHFX to normo- and moderate hypofractionation have shown that > G2 gastrointestinal (GI) toxicity is nearly absent, while acute and late GU toxicity is higher compared to the normo- or moderately hypofractionated arms [1–4]. In contrast to the rectum, where spacers can be used to separate the organ at risk from the target volume [5–7], such strategies are not possible for the urethra, which is embedded within the clinical target volume (CTV).

Patients of the CHHiP trial cohort were subjected to a secondary analysis of α/β ratios for dysuria and hematuria endpoints and these were calculated to be 0.6 Gy to 2.0 Gy, which is lower than expected from typical late α/β ratio assumptions of 3.0 Gy to 5.0 Gy [8]. This suggests a lower therapeutic gain for GU toxicity by hypofractionation. The specific anatomical structure responsible for radiation-induced GU toxicity is still a topic of controversy. However, some authors have proposed the urethra and bladder trigone as potential contributors [9–11].

Therefore, urethra steering (i.e., avoiding dose maxima within the urethra) or urethra sparing (i.e., dose reduction to the urethra) has been proposed in order to mitigate urethral toxicity [12]. However, on planning and cone-beam CT (CBCT), the urethra is not visible, and it can even be difficult to delineate on MRI. In addition, it is not known whether the urethra shape within the prostate remains constant over the course of the treatment period. Prostate rotations can be significant, suggesting that the urethral shape (bending) may change over time.

This introduces considerable uncertainty to the concept of urethra sparing because it is not known whether the favorable dose distribution of the planning CT can actually be reproduced during treatment in a real-world scenario.

A key aspect of UHFX of the prostate is intrafractional motion control, which is considered obligatory in order to ensure that prostate movement can be compensated for during fractions lasting longer than 3 min [13]. We employed continuous position monitoring using an electromagnetic transmitter placed into the prostatic urethra using a Foley catheter [14]. Besides real-time motion control during beam delivery, this approach allows clear visualization of the urethra in both the planning CT and CBCTs acquired before and during each fraction.

This allowed us to assess the dose coverage of the CTV and the urethra in all fractions of the 12 study patients. We also analyzed how changes in urethra shape and prostate movement impacted the robustness of urethral sparing and CTV coverage.

## Methods

Prior to treatment planning, an MRI-compatible RayPilot ViewCath catheter (Micropos Medical™, Gothenburg, Sweden) was inserted into the prostatic urethra, and the blocking balloon was filled with 18 ml of distilled water and 2 ml of contrast agent (Visipaque^TM^) to improve visibility on CT imaging. For both CT and MRI, the bladder was filled with 125 ml of saline. The ViewCath catheter was removed immediately after imaging.

**Fig. 1:**
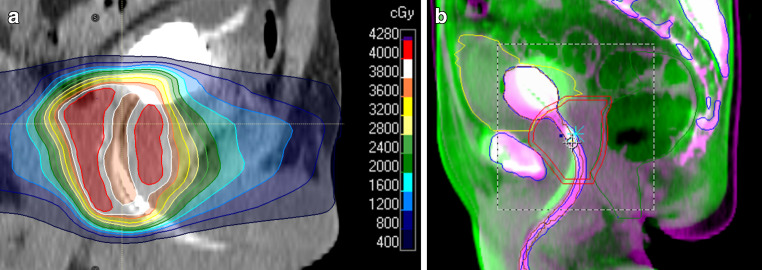
**a** Dose distribution as planned and overlay of cone-beam CT (green) and **b** planning CT (magenta) of an exemplary patient, registered to the transmitter

The planning risk volume (PRV) urethra was contoured based on the outline of the catheter on the planning CT using a 7-mm diameter brush, accounting for the catheter diameter of 5.2 mm and the urethral wall. Treatment planning was based on the PACE‑B study protocol, with the planning target volume (PTV) receiving five times 7.25 Gy and the CTV receiving five times 8.00 Gy. The PTV was defined by expanding the CTV by 3 mm posteriorly and 4 mm in all other directions. Subsequently, the PRV with an additional 2‑mm margin was subtracted from the CTV to generate a technical CTV. In the following, this structure is referred to as the CTV for simplicity. Urethral sparing was performed by limiting the near-maximum dose (D1) of the PRV to 38.00 Gy and the near-minimum dose (D99) to 36.25 Gy (see Fig. 1 for an exemplary plan).

Treatment plans were generated using RayStation (RaySearch Laboratories, Sweden) using volumetric arc therapy (VMAT) with two arcs and a 6-MV flattening filter-free (FFF) technique. Patients were treated on an Elekta LinAc (Elekta, Sweden) with an Agility head with 5 mm leaf width and a Precise table with four degrees of freedom.

Prior to the first fraction, a RayPilot HypoCath catheter (Micropos™, Sweden) was inserted, and the blocking balloon was filled with diluted contrast agent. The RayPilot system was used for tracking prostate motion in real time, as previously described [14], with a 3-mm interruption threshold chosen arbitrarily based on our experience that even smaller thresholds lead to an excessive number of treatment interruptions.

An initial CBCT was performed and registered using the clearly visible transponder of the HypoCath catheter (Fig. 1a).

When prostate motion exceeded the predefined 3‑mm threshold in the lateral, vertical, or longitudinal direction during irradiation, treatment delivery was interrupted, and a new image-guided radiation therapy (IGRT) acquisition was initiated. This procedure was repeated as necessary until completion of the treatment. However, these corrections were not considered in the dose calculation related to intrafractional prostate motion in the following.

**Fig. 2:**
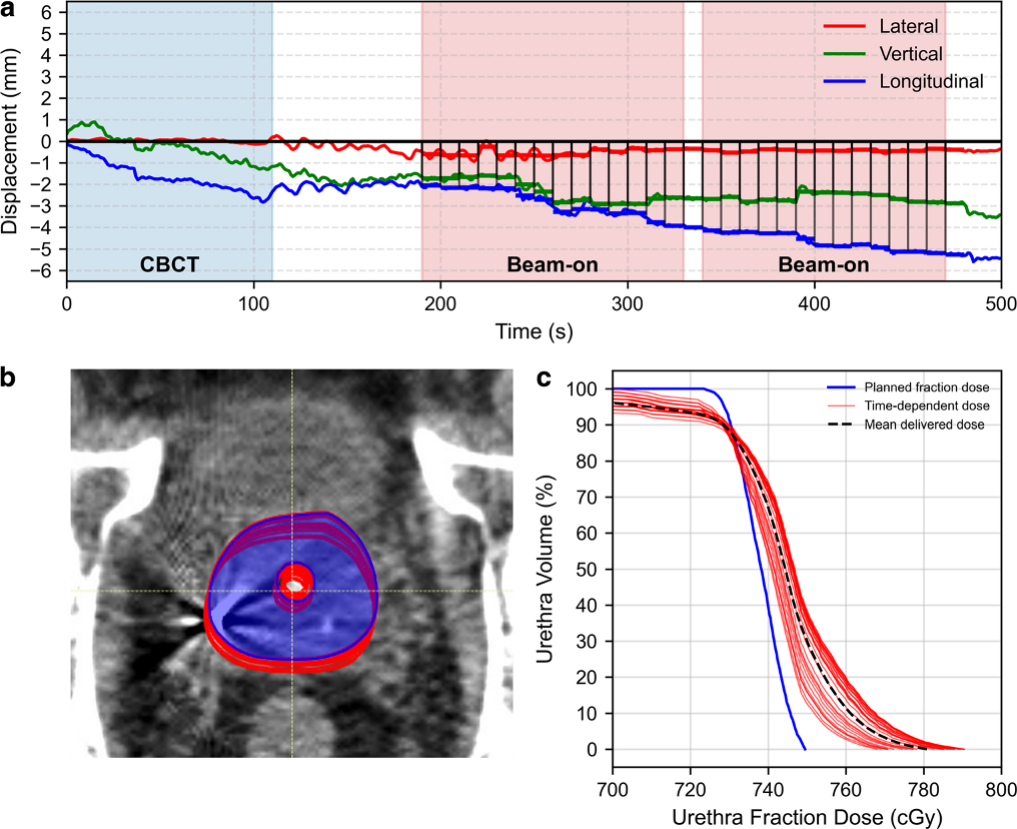
**a** Exemplary treatment fraction. Blue shaded area represents the time required to acquire the cone-beam CT (CBCT), which takes 110 s for our prostate preset. The following 80 s are used for matching and correcting the patient position. The red shaded area represents beam-on time (two arcs). Vertical black lines during beam-on time illustrate the 10-second mapping timepoints used for cumulative dose calculations. **b** Different positions of the prostate during treatment delivery. Each red contour represents the mean of a 10-second interval during beam-on time. The blue shaded area is the initial position. **c** Urethra dose–volume histogram as a function of time. Each thin red line represents a different prostate position during the fraction. The bold blue line is the planned fraction dose; the black dashed line is the mean delivered dose

The average duration of a fraction, excluding interruptions, was 7:50 min (standard deviation 1 min 36 s) from the initial CBCT to the end of treatment, with a beam-on time of 4:30 min. Figure 2a shows prostate movement over time for an exemplary treatment fraction.

The dosimetric effects of urethral bending and prostate motion during irradiation were systematically evaluated. A synthetic CT was generated from the initial CBCT for each treatment fraction, which was then used to compute an adapted dose distribution while keeping the plan as-is. These adapted dose distributions, reflecting the patient’s anatomical configuration at the start of each fraction, enabled a detailed assessment of the impact of interfractional prostate motion on treatment accuracy and dose distribution.

To simulate intrafractional prostate movement, prostate motion data from 60 individual treatment sessions analyzed in our previous study [14] were mapped one-to-one to the 60 fractions analyzed in the present study. For each fraction, the motion traces were subdivided into successive 10-second segments and the average displacement along each spatial axis was determined. These shifts were then incorporated into the treatment planning system by adjusting the positions of the CTV and PRV to estimate the corresponding time-dependent delivered dose. Figure 2b illustrates how changes in prostate positions during treatment delivery translate into corresponding variations in dose distribution.

For each individual prostate position, the dose statistics for the PRV and the CTV were calculated (Fig. 2c). The mean values of those time-dependent doses were calculated to assess the effect of intrafractional variability on dose distributions over the course of each fraction (Fig. 2d).

## Results

A total of 60 fractions performed on 12 patients treated between January 2024 and February 2025 were analyzed.

Interfractional differences in urethral shape and curvature were clearly visible in sagittal CBCT views during the initial IGRT of each fraction (Fig. 1b).

During the pretreatment planning phase, urethral sparing using dose objectives of D1 38.00 Gy and D99 36.25 Gy was achieved in all patients, with a mean PRV D1 of 37.64 Gy and a mean D99 of 36.25 Gy across the cohort. The CTV was planned with a mean median dose of 40.66 Gy.

**Fig. 3:**
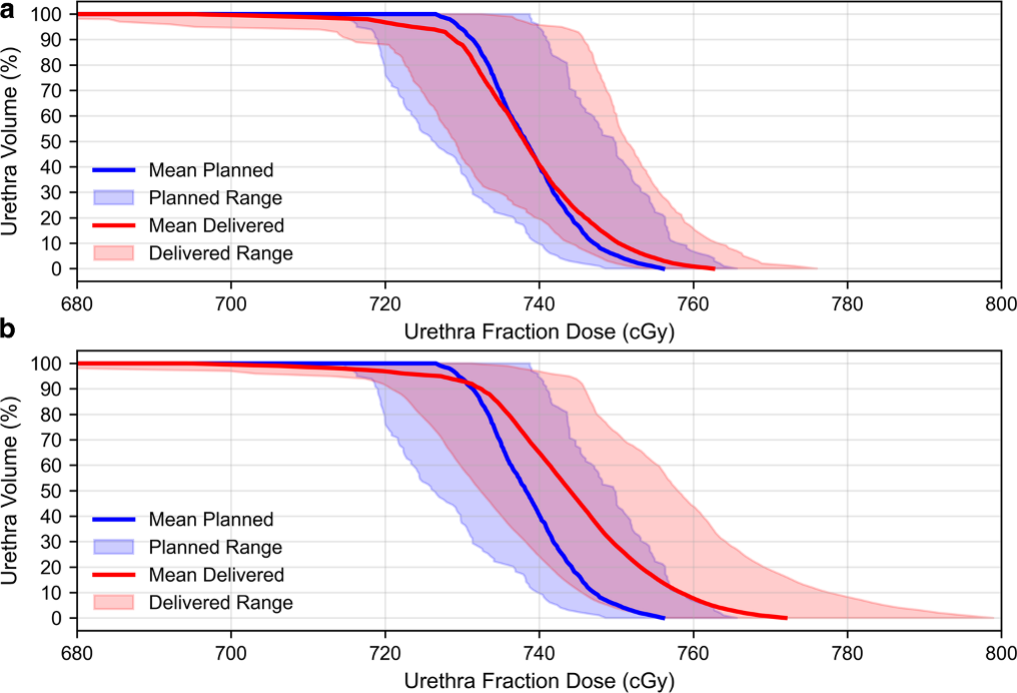
**a** Mean urethra dose–volume histogram (DVH) of planned and delivered doses with respect to changes in urethral shape and **b** the combined effect of shape and translatory motion. The blue and red lines represent the mean planned and delivered doses, respectively. Shaded areas show the corresponding fraction-dependent DVH range

Considering only interfractional effects, specifically changes in urethral curvature, the PRV D1 increased by 0.3 Gy (+0.8%) and D99 decreased by 1.2 Gy (−3.3%; Fig. 3a).

The combined effect of interfractional urethral shape changes and intrafractional prostate movement over the course of all fractions was an increase in the planned D1 of the PRV of 0.8 Gy (+2.1%) and a decrease in the planned D99 of the urethra of 1.4 Gy (−3.9%; Fig. 3b).

The fraction associated with the largest dose deviation showed an increase in urethral D1 from 7.48 to 8.11 Gy. The highest cumulative increase in urethral D1 for a single patient over the course of a full treatment was 1.94 Gy (+5.2%).

**Fig. 4:**
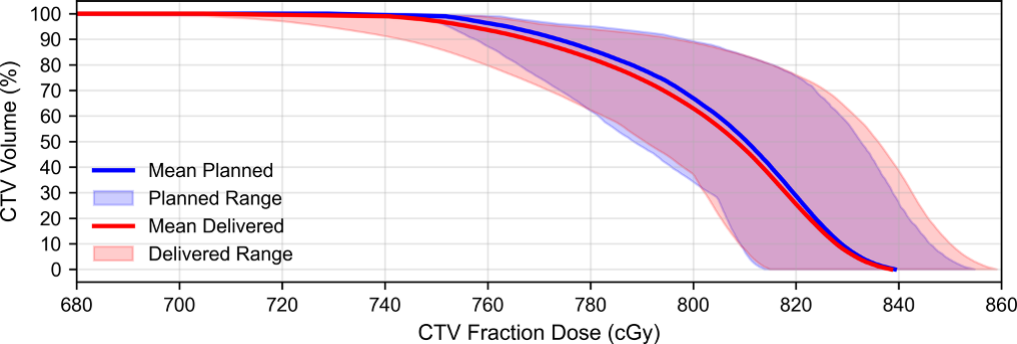
Mean clinical target volume (*CTV*) dose-volume histogram. The blue and red lines show the mean planned and mean delivered dose, respectively. Shaded areas represent the corresponding fraction-specific dose range

The impact of urethral shape changes and the combined effect of prostate motion on the median CTV dose was a mean decrease of 17 cGy (−0.4%) for all patients (Fig. 4). For the patient exhibiting the largest prostate motion, the overall D95 decreased from 37.9 to 36.4 Gy (−4.0%).

## Discussion

In this study, we investigated the clinical feasibility and dosimetric robustness of urethra-sparing radiotherapy by retrospective analysis of dose distributions on CBCT images. Our goal was to assess the impact of inter- and intrafractional anatomical variations, i.e., changes in urethral curvature as well as prostate motion, on the actual delivered dose to the CTV and the urethra.

The use of a contrast-enhanced HypoCath catheter allowed for clear urethral visualization in both the planning CT and CBCT, enabling accurate dose recalculation and verification.

We analyzed motion-related dosimetric changes (intrafractional effects) and anatomically related changes (interfractional effects) separately. While interfractional effects were negligible (+0.8% PRV D1), intrafractional changes were more pronounced, despite patient preparation aimed at minimizing prostate motion, including bowel emptying prior to treatment and a brief rest period on the treatment couch before IGRT acquisition and initiation of prostate motion tracking. For example, one patient experienced a substantial intrafractional PRV D1 increase of 5.2%, thus mitigating the urethra-sparing effect substantially, given the dose difference between the prescribed PRV D1 (38.0 Gy) and the CTV median dose (40.0 Gy) is 5.3%.

Considering the CTV, inter- and intrafractional variability is not as significant as for the PRV, because the CTV has a much larger volume compared to the urethra and sufficient margins. Therefore, the median dose responds less sensitively to variations in urethra shape or prostate motion.

However, our results show that translatory intrafractional movement is strongly dependent on the duration of the total treatment, assuming that no motion compensation is carried out during treatment. Treatment duration varies according to many factors, such as the IGRT method, radiation technique (step-and-shoot IMRT, VMAT, FFF, etc.), and patient-related factors. Therefore, our results cannot be universally applied but must be interpreted in the context of actual treatment times. Our results underscore the importance of short fraction times, to which the duration of CBCT, the time for matching, and beam-on time contribute. The residual uncertainty should be compensated for by adequate individualized and time-dependent PTV margins, as proposed by Janssen et al. [15]. Universal margin recipes such as the widely used Van Herk formula [16, 17] are inadequate, since they are designed for normofractionated regimens and cannot account for varying fraction duration and systematic target drift.

In contrast, we demonstrated that the isolated impact of anatomical variations in urethra shape, i.e., the curvature of the urethra, is negligible. Although these variations, which may stem from prostate and/or hip rotations or different bladder fillings, appear significant during visual inspection of CBCT images, they had only minimal impact on the robustness of urethra sparing. These findings suggest that a similar robustness may also be expected in the context of a simultaneous integrated boost on the MRI- and/or PET-CT-dominant lesions, which is a promising strategy for high-risk patient cohorts [18, 19].

The impact of prostate motion on target volume coverage was comparable to previously reported motion-related effects under similar intrafractional motion-control strategies [14]. These findings demonstrate that urethra sparing can be implemented without compromising target coverage, as long as appropriate intrafractional motion control and treatment times are in place.

A key strength of our study is the direct visualization of the urethra during the planning CT and IGRT CBCTs combined with real-time position tracking, which enabled highly accurate assessment of dose coverage over the course of UHFX treatment. Limitations are the lack of generalizability of our data with respect to treatment duration, setup error, and intrafractional motion management, which may differ between radiation therapy facilities and workflows. Future studies will need to show that urethra sparing translates to improved GU toxicity, as predicted by NTCP modelling [20].

## Conclusion

Urethra sparing is feasible and only marginally impaired by variations in urethral shape and bending, but it is sensitive to longer treatment durations due to intrafractional motion. Direct visualization of the urethra before each fraction is not necessary for image-guided radiation therapy (IGRT), but intrafractional motion control is recommended.
